# Canonical Wnt Signaling Pathway on Polarity Formation of Utricle Hair Cells

**DOI:** 10.1155/2021/9950533

**Published:** 2021-05-22

**Authors:** Di Deng, Xiaoqing Qian, Binjun Chen, Xiaoyu Yang, Yanmei Wang, Fanglu Chi, Yibo Huang, Yu Zhao, Dongdong Ren

**Affiliations:** ^1^Department of Otolaryngology, Head and Neck Surgery, West China Hospital, Sichuan University, Chengdu 610041, China; ^2^NHC Key Laboratory of Hearing Medicine (Fudan University), Shanghai 200031, China; ^3^ENT Institute and Department of Otorhinolaryngology, Eye & ENT Hospital, Fudan University, Shanghai 200031, China

## Abstract

As part of the inner ear, the vestibular system is responsible for sense of balance, which consists of three semicircular canals, the utricle, and the saccule. Increasing evidence has indicated that the noncanonical Wnt/PCP signaling pathway plays a significant role in the development of the polarity of the inner ear. However, the role of canonical Wnt signaling in the polarity of the vestibule is still not completely clear. In this study, we found that canonical Wnt pathway-related genes are expressed in the early stage of development of the utricle and change dynamically. We conditionally knocked out *β*-catenin, a canonical Wnt signaling core protein, and found that the cilia orientation of hair cells was disordered with reduced number of hair cells in the utricle. Moreover, regulating the canonical Wnt pathway (Licl and IWP2) in vitro also affected hair cell polarity and indicated that Axin2 may be important in this process. In conclusion, our results not only confirm that the regulation of canonical Wnt signaling affects the number of hair cells in the utricle but also provide evidence for its role in polarity development.

## 1. Introduction

The cochlea and vestibule are important for hearing and balance, respectively. They detect sound and position signals through stereociliary bundle on the surface of hair cells (HCs) [1–4]. The vestibular system contains utricular maculae, saccular maculae, and three semicircular canal cristae. Planar polarity of the stereociliary bundle guarantees that the range of motion is detected properly by vestibular HCs [5]. In the inner ear, the stereocilia are arranged in an ascending cluster, with the highest being located adjacent to the kinocilium [6, 7]. The direction of stereociliary bundle between adjacent HCs are highly similar. Compared to the cochlea, an artificial line (line of polarity reversal, LPR) passes through the maculae. The maculae are divided into two regions: the kinocilium of the utricular maculae faces the LPR, while the kinocilium of the saccular maculae points away from the LPR [8].

**Figure 1:**
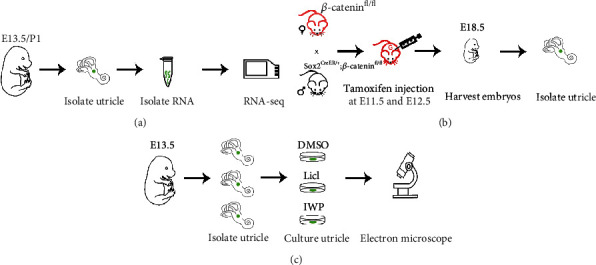
The experimental procedure. (a) RNA-sequencing of the utricle of E13.5 and P1 mice. (b) To conditionally knockout *β*-catenin, the *β*-catenin^f/f^ female mice were mated with *β*-catenin^f/f^ male mice with Sox2^CreER+^; the pregnant mice were treated with tamoxifen injection at E11.5 and E12.5, and the utricle of the embryonic mice was harvested at E18.5. (c) Utricles of E13.5 mice were cultured *in vitro* with DMSO (control), LiCl (activator), and IWP2 (inhibitor).

In the canonical Wnt pathway, Wnt protein binds to Frizzled on the cell surface to inhibit the activity of the *β*-catenin-degrading complex composed of GSK-3, APC, and Axin. *β*-Catenin accumulates in the cytoplasm and is then transferred to the nucleus, where it controls embryogenesis and cell fate through T-cell factor/lymphoid enhancer-binding factor (TCF/LEF). The canonical Wnt signaling pathway and its downstream target genes play an important regulatory role in cell proliferation [9–13] and differentiation [14–18] during inner ear development [19], as well as in the survival of HCs [20–24] and spiral ganglion neurons [25]. During development, the expression of Wnt signaling significantly decreased with age in the inner ear [26]. The canonical Wnt pathway is essential to induce Pax2-positive otic placode cells to form an auditory sac [27]. In our previous studies, we found that polarity of striola in the medial region occurred earlier than in the lateral region, consistent with proliferation and differentiation in utricle HCs [28]. This led us to hypothesize that the canonical Wnt signaling pathway is involved in polarity development in the utricle. Here, we found differences in the expression of Wnt pathway-related genes among different stages of polar formation in the utricle. We conditionally knocked out *β*-catenin, a core protein in the canonical Wnt signaling pathway, in Sox2-positive cells in mice. The number and polarity of HCs were also affected. Moreover, the polarity of the utricle was affected by treatment with an activator or inhibitor of the canonical Wnt pathway *in vitro*, and we found that Axin2 may be a key role in this process (Figure 1).

## 2. Materials and Methods

### 2.1. Animal Protocols

All protocols were approved by the Animal Care and Use Committee of the EENT Hospital of Fudan University. Healthy wild-type C57BL/6 mice were purchased from Shanghai SLAC Laboratory Animal Co., Ltd. (Shanghai, China). Female and male mice were mated in the same nest at 10 AM and recorded as E0. They were separated at 10 AM the next day and recorded as E0.5. In turn, E11.5 were recorded on day 12, E12.5 on day 13, and E13.5 on day 14. We also performed tap detection at E0.5.

Sox2^CreER^ and Ctnnb1 ^flox(exon2-6)^ mice were provided by Huawei Li at Fudan University and maintained in a mixed background of C57BL/6J and BALB/C. Standard PCR was performed to genotype transgenic mouse offspring. DNA was isolated by incubating the tail tip in a 100 *μ*L mixture of Direct PCR and Protein K (100 : 2) at 55°C overnight and then at 95°C for 1 h. The primers used were as follows: GA486 (TGCCACGACCAAGTGACAGCAATG) and GA487 (ACCAGAGACGGAAATCCATCGCTC) for the Cre allele, with an expected fragment size of 300–400 bp; R15 (AAGGTAGAGTGATGAAAGTTGTT); and R16 (CAC CAT GTC CTC TGT CTA TTC) for the *β*-catenin knockout allele, with expected fragment sizes of ~300 and 223 bp for the mutant and wildtype, respectively. To activate Cre and avoid premature abortion due to tamoxifen, a tamoxifen/progesterone mixture dissolved in corn oil (Sigma Aldrich, St. Louis, MO, USA; 1 mL corn oil : 10 mg tamoxifen : 20 mg progesterone) was injected intraperitoneally once a day for 2 consecutive days. The dose on the first day (E11.5) was 0.1 *μ*g/g tamoxifen, and the dose was halved on the following day (E12.5).

### 2.2. Utricle Harvest

The pregnant mice were anesthetized and killed. The embryo were removed from the uterus and quickly placed into a cold solution of 1× phosphate-buffered saline (PBS; pH = 7.4). The utricle was dissected with the microscope. P1 mice were anesthetized and killed; then, the utricle was harvested, and the otolith was removed with the microscope and harvest the utricle.

### 2.3. Tissue Culture *In Vitro*

The freshly dissected utricle was placed in Dulbecco's modified Eagle's medium (DMEM)/F12 medium (20% foetal bovine serum [FBS], B27 (1 : 100), streptomycin [100 U/mL]). The Petri dishes were incubated at 37°C, with a concentration of 5% CO_2_ and humidity of 95%. Dimethyl sulfoxide (DMSO), lithium chloride (LiCl), or IWP2 was added 24 hours later. We treated cultures in the activator group with the canonical Wnt pathway activator LiCl (2.5 *μ*M/mL). The canonical Wnt inhibitor IWP2 (1.0 *μ*M/mL) was added to the inhibitor group. DMSO (1.0 *μ*M/mL) was added to the control group. The final culture concentration depended on the growth of the utricle. The culture medium was replaced in full every 24 hours, and the tissue was removed and fixed on the day 5 after treatment. For all experiments, three biological replicates were used.

### 2.4. PCR and RT-qPCR

The reaction protocol for PCR was as follows: predenaturation at 94°C for 5 min, 32 cycles of denaturation at 94°C for 1 min, annealing at 55°C for 1 min, elongation at 72°C for 1.5 min, and a final elongation at 72°C for 10 min.

RNA was extracted from the utricle using the RNeasy Micro Kit (Qiagen, Hilden, Germany), and cDNA was synthesized using the GeneAmp® PCR System 9700 (Applied Biosystems, Foster City, CA, USA) by reverse transcription: 42°C for 15 min and 85°C for 5 s. The qPCR reaction protocol was as follows: 94°C for 30 s, followed by 45 cycles at 94°C for 5 s and 60°C for 30 s. Each sample was run in triplicate for analysis purposes. At the end of the PCR cycles, melting curve analysis was performed to validate the generation of the expected PCR product. The primer sequences were designed in the laboratory and synthesized by TsingKe Biotech (Beijing, China) based on the mRNA sequences obtained from the NCBI database, as follows:

GADPH (F: GCAAGGACACTGAGCAAGA; R:GGATGGAAATTGTGAGGGAG), Apc (F: GCCTGGATGAGCCATTTATAC; R: AGTTTCATTCCCATTGTCGT), Wif1 (F: TTGTACCTGTGGATCGACG; R:GGCTTTCCTGAAATCATGTGT), Camk2d (F: AGATCAAGGCCGGAGCTTA; R:CAGAGGCTGTGATACGTT), Fzd6 (F: CTTCAGTGGCCTGTATCTT; R:CCATGTCATCTCCCAGGT), Axin2 (F:AGAAGAGGAGTGGACGTGTG; R:AGCTGTTTCCGTGGATCTCA), Vangl2 (F:TCTCTGGCCCTGACACATTT; R:ACTGAGGAAGAGGGGAGACT), Prickle2 (F:ATGCCACCTTCTTCCTCCTC; R:AGTAGGTGACAAATGGCCGA).

### 2.5. RNA-Sequencing (RNA-Seq) and Protein-Protein Interaction (PPI) Network

Total RNA was extracted from the utricle (E13.5 and P1,wild-type C57BL/6 mice) and then submitted to Otogenetics Corporation (Atlanta, GA, USA) for RNA-Seq assays. We assessed RNA integrity and purity using an Agilent Bioanalyzer (Agilent Technologies, Santa Clara, CA, USA). cDNA was generated from total RNA using the Clontech SMARTer PCR cDNA Synthesis Kit (Clontech Laboratories, Inc., Mountain View, CA USA, catalogue #634891). Bioruptor (Diagenode, Inc., Denville, NJ, USA) was used to fragment cDNA, an Agilent Bioanalyzer was used for profiling, and SPRIworks HT (catalogue #B06938; Beckman Coulter, Pasadena, CA, USA) was used to prepare the Illumina library. The quality, quantity, and size distribution of the Illumina library were determined using TapeStation (Agilent Technologies).

The DAVID database (https://david.ncifcrf.gov/) is a commonly used database for gene analyses. KEGG pathway enrichment analyses were performed using DAVID, and the TXT files of the results were downloaded for subsequent analysis. For the Bonferroni test, *P* values < 0.05 were set considered significant, and Log2 (fold_change) values between -1 and 1 were excluded from the analysis. STRING (https://string-db.org/) was used to analyse the interactions between proteins. Many signaling pathways play important roles in the development of the utricle. To better understand the role of the Wnt signaling pathway, we only included the Wnt pathway-related genes in this analysis.

### 2.6. Electron Microscope

Utricles were fixed with 2.5% glutaraldehyde at 4°C overnight and then processed using 2% tannin and 1% osmium acid. Graded ethanol series were used to dehydrate the samples, and liquid CO_2_ (EM CPD300; Leica, Wetzlar, Germany) was used to dry them. An E-1045 sputter coater (Hitachi, Tokyo, Japan) was used to coat specimens with 100 Å Au. A NOV A NanoSEM 230 scanning electron microscope (FEI, Hillsboro, OR, USA) was used to scan the samples.

### 2.7. Whole-Mount Immunostaining

Mouse embryos were harvested at E18.5. Otocysts were dissected in cold PBS and fixed with 4% paraformaldehyde (Electron Microscopy Services, Hatfield, PA, USA) for 1 h at 4°C. After washing with PBS, utricles were dissected and blocked with 10% donkey serum, and 0.1% Triton-X in PBS for 30 min at room temperature, followed by incubating with primary antibodies diluted in PBS containing 5% donkey serum and 0.1% Triton-X overnight at 4°C. The next day, after washing extensively with PBS, tissues were incubated with secondary antibodies at 1 : 1,000 dilution in 0.1% Triton-X100 (in PBS) for 2 h at room temperature. After extensive PBS washing, samples were mounted in antifade Fluorescence Mounting Medium (Agilent Technologies) on coverslips. We used mouse anti-*β*-spectrin (1 : 500; catalogue #612562; BD Biosciences) primary antibody and Alexa Fluor 568 Donkey Anti-Mouse IgG (1 : 1,000; Invitrogen, Carlsbad, CA, USA) secondary antibody. FITC-conjugated phalloidin (1 : 1,000; Invitrogen) was used to visualize actin in the stereocilia of sensory HCs.

### 2.8. Quantification and Statistical Analyses

**Figure 2:**
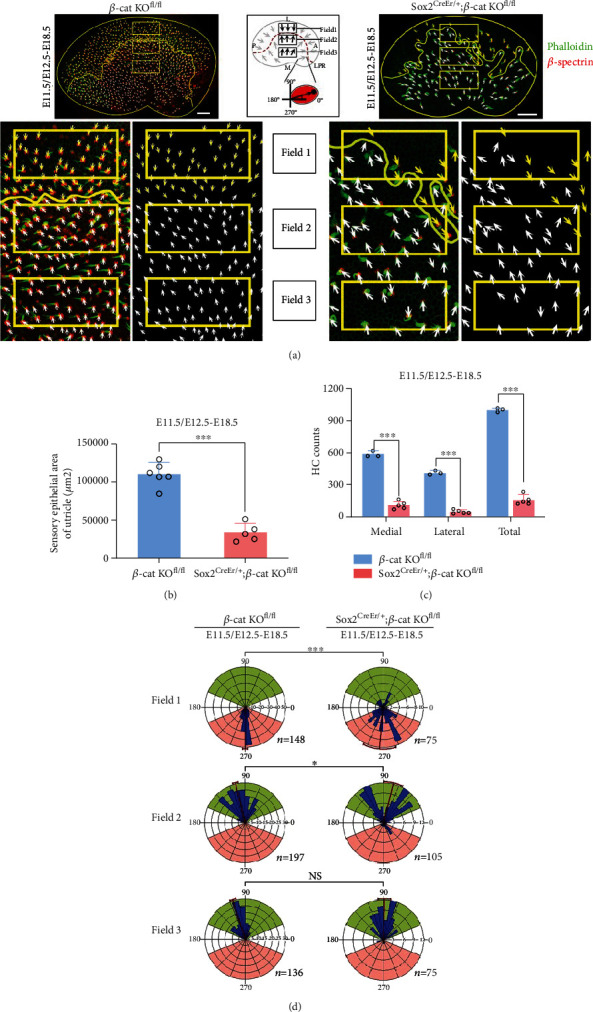
Knockout (KO) *β*-catenin in Sox2 positive cells influenced the orientation and number of vestibular hair cells. (a) Compared to the controls (left), Sox2^CreER/+^ and *β*-catenin^fl/fl^ (tamoxifen given at E11.5 and E12.5) utricles (right) showed a significant decrease in sensory epithelium area and HC number and a different extent of hair bundle orientation irregularity in fields 1–3. The schematic diagram in the middle black box defining regions of the utricle and illustrating the hair bundle polarity pattern. (b) Quantification of the sensory epithelium area of control and *β*-catenin KO utricles (controls, *n* = 6; mutants, *n* = 5). (c) Quantification of HC density on the lateral and medial sides of the LPR and the whole sensory epithelium, from control and *β*-catenin KO utricles (controls, *n* = 3; mutants, *n* = 5). (d) Circular histograms of the hair bundle orientation in field 1, field 2, and field 3 for control and *β*-catenin KO utricles (controls, *n* = 3; mutants, *n* = 5). LPR: line of polarity reversal; L: lateral; M: medial; A: anterior; P: posterior. ^∗^*P* < 0.05, ^∗∗∗^*P* < 0.001.

Whole-mount immunostaining images were acquired using a Zeiss LSM800 confocal microscope (Zeiss, Oberkochen, Germany) at 40× magnification, and the whole utricle image was obtained by flattening the overlapping parts of original images using Photoshop CS4 (Adobe Systems). The overall outline of a utricle was drawn on the stitched confocal image, and the area of the sensory epithelia was measured using the Zen image processing system (Zeiss). The number of HCs on the composite image was counted manually using Photoshop CS4 (Adobe Systems). Cell number was scored in two regions divided by LPR, defined as lateral and medial region. Hair bundle orientation was scored in three 40 × 80 *μ*m^2^ regions in the middle of the utricle, defined as field 1, field 2, and field 3 (Figure 2(a)). The hair bundle angle was measured based on the position of kinocilium (spectrin-negative on the apical surface), by defining 0° as the anterior apex and 90° as the lateral side, and quantified using ImageJ64 (National Institutes of Health, Bethesda, MD, USA). A single kinocilium could be found directly in the electron microscope specimens. Matlab and Screen protractor were used to draw the angle distribution diagram. Statistical analyses were conducted using Prism 8 (GraphPad Software Inc., San Diego, CA, USA). A two-tailed, unpaired Student's *t*-test was used to determine statistical significance. *P* < 0.05 was considered significant. Data are shown as mean ± SD.

## 3. Result

### 3.1. Wnt Signaling Is Active in the E13.5 Utricle

Our previous studies showed that E13.5 is a critical period for the formation of polarity in the utricle [28]. Two clusters of HCs with opposite cilia began to appear in the utricle, gradually forming LPR. The Wnt signaling pathway is important for embryonic development and plays a variety of roles at different time points during development. Therefore, the primary purpose of our study is to understand the expression of Wnt pathway-related genes in the early utricle.

**Figure 3:**
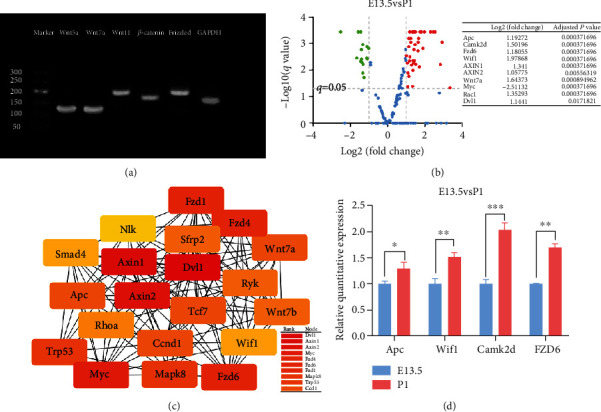
Wnt signaling pathway is active in the E13.5 utricle and changes during utricle development. (a) RT-PCR shows that Wnt5a, Wnt7a, Wnt11, *β*-catenin, and Frizzled were expressed in the utricle of E13.5 mice. (b) Differentially expressed Wnt pathway-related genes between the E13.5 and P1 utricle. The red dots represent the upregulated genes based on an adjusted *P* value < 0.05 and ∣log2 (fold change) | >1; the green dots represent the downregulated genes based on an adjusted *P* value < 0.05 and ∣log2 (fold change) | >1; the blue spots represent genes with no significant difference in expression. (c) PPI network of E13.5 and P1 utricle. Circles represent genes. Lines represent interactions between gene-encoded proteins, and the top 10 rankings are listed. (d) The relative levels of APC, Wif1, Camk2d, and Fzd6 were analysed by RT-qPCR. The data presented are based on at least three independent experiments. The data are presented as the mean ± SEM. ^∗^*P* < 0.05, ^∗∗^*P* < 0.01, ^∗∗∗^*P* < 0.001.

Wnt5a, Wnt7a, Wnt11, *β*-catenin, and Frizzled are important genes in the canonical/noncanonical Wnt signaling pathway. We detected these genes in the utricle by RT-PCR. Our research shows that these genes were expressed in the utricle of E13.5 mice (Figure 3(a)).

### 3.2. Changes of Wnt Signaling in the Early Development of the Utricle

To explore changes in Wnt signaling pathway activity during early development of the utricle, RNA from the utricle of P1 and E13.5 was extracted for RNA-Seq analysis.

Our results showed that Wnt pathway-related genes were differentially expressed. Compared to E13.5,the upregulated genes in P1 were Apc, Axin1, Axin2, Camk2d, Rac1, Fzd1, Fzd6, Dvl1, Wif1, Smad4, Wnt7a, Nkd1, Sfrp2, Ryk, Siah1a, Camk2g, Ctnnd2, Rspo3, Ppp3cb, Map3k7, Tcf7l1, Prkacb, Porcn, Cxxc4, Prkaca, Vangl1, Ppp3ca, Senp2, Prickle2, Nfatc1, Nfatc3, Nlk, Fzd4, Rock2, Prkcd, Prkce, Prkcz, Ctbp2, Prkci, Bambi, Mapk8, Csnk2a2, Frat1, Prickle1, Ppard, Gsk3a, Tle1, Tle4, Gpc1, Pygo2, Sdc3, and Sdc4. The downregulated genes included Camk2b, Psen1, Ruvbl1, Trp53, Ccnd1, Cacybp, Rhoa, Sfrp5, Csnk1g1, Csnk2b, Sdc1, Bcl9, Gpc2, Gpc3, Lgr5, Ccnd2, Cby1, Dkk2, Rbx1, Myc, Wnt7b, Ppp3r1, Frat2, Serpinf1, and Rhoc (Figure 3(b)). There were some representative genes of canonical Wnt signaling, such as Apc, Axin2, and Wif1. The protein interaction network of Wnt pathway-related genes showed their ranking (Figure 3(c)). Finally, we used RT-qPCR for verification and found that some canonical Wnt pathway-related genes differed in the expression levels of E13.5 and P1 (Figure 3(d)).

### 3.3. Conditional Knock-out *β*-Catenin Affects the Number and Polarity of Vestibular HCs

The areas of utricle maculae in the control and experimental groups were 96,326.63 ± 36,029.13 *μ*m^2^ (*n* = 7) and 33,824.00 ± 10,515.93 *μ*m^2^ (*n* = 5), respectively, *P* < 0.05 (Figure 2(b)). In the control group (*n* = 3), the total number of HCs was 995.67 ± 14.61, with 585.33 ± 33.40 and 410.33 ± 15.41 in the medial and lateral regions, respectively, whereas in the experimental group (*n* = 5), the total number of HCs was 156.80 ± 40.09 with 109.00 ± 30.25 and 47.80 ± 15.46 in the medial and lateral regions, respectively, *P* < 0.05 (Figure 2(c)). As in the control group, *β*-catenin knockout mice still showed relatively clear LPRs, with the HCs on both sides having roughly normal directions (Figure 2(a)). However, it is worth noting that the direction of some HCs near LPR, in filed 1 and filed 2 regions, was disordered (Figure 2(d)).

### 3.4. Regulation of Wnt Signaling *In Vitro* Leads to Polarity Changes in the Utricle

To determine the role of the Wnt signaling pathway in the polarity of HCs in the utricle, we regulated Wnt signaling pathway activity *in vitro* using a Wnt/*β*-catenin inhibitor (IWP2) and a Wnt/*β*-catenin activator (LiCl) [29]. According to daily observations of the growth of the utricle, the final culture concentrations were 2.5 and 1.0 *μ*M/mL for LiCl and IWP2, respectively.

**Figure 4:**
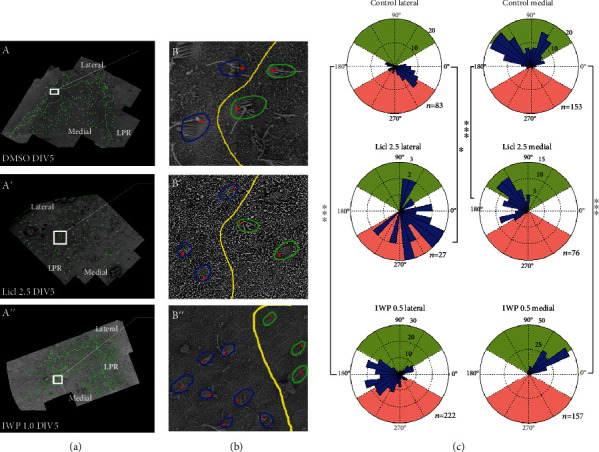
Regulation of Wnt signaling *in vitro*. (a) A' and A” correspond to the control, activation, and inhibition groups, respectively. DMSO, LiCl, and IWP2 were added into Petri dishes with E13.5 utricles and cultured for 5 days. Scanning electron microscopy showed the polarity and line of polarity reversal (LPR) of the hair cells (HCs) in the control group. The green line represents the LPR, and the green arrow represents the direction of the HC polarity. (b) B' and B” are the white boxes in (a) A' and A,” respectively; the green circles represent the HCs on the medial side of the LPR, and the blue circles represent the HCs on the lateral side of the LPR; the red dots represent the position of the kinocilium. Circular histograms of the hair bundle orientation in lateral and medial regions for the control, activator, and inhibitor utricles. ^∗^*P* < 0.05, ^∗∗∗^*P* < 0.001.

Using a scanning electron microscope, we found that the polarity of the utricle was affected by treatment with the inhibitor or activator. Compared to the control group (Figures 4(a)and 4(b)), the position of the LPR in the activation group was changed (Figure 4, A' and B'), and the polarity of HCs on the lateral and medial side of the LPR was disturbed after adding the activator. In the inhibition group, the polarity of HCs on the lateral and medial sides of the LPR was disturbed (Figure 4, A” and B”). Circular histograms of the hair bundle orientation also showed the difference (Figure 4(c)).

### 3.5. Axin2 Is Decreased in Both the Activation and Inhibition Groups in mRNA

**Figure 5:**
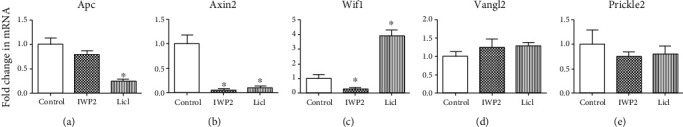
Analysis of Wnt pathway-related genes in vitro treatment at RNA level. (a) Compared with the control group, the expression of Apc was decreased with Licl treatment, and there was no statistical difference with IWP2 treatment. (b) The expression of Axin2 was decreased with IWP2 treatment or Licl treatment. (c) The expression of Wif1 was decreased with IWP2 treatment and was increased with Licl treatment. (d, e) Vangl2 and Prickle2 were no statistical difference with IWP2 treatment or Licl treatment, compared with the control group. ^∗^*P* < 0.05.

According to RNA-seq results and common Wnt pathway genes, RT-qPCR was performed to detect canonical Wnt signaling pathway-related genes (Apc, Wif1, and Axin2) and PCP core genes (Prickle2 and Vangl2) in cultured utricle in vitro to explore the possible mechanism of polarity change (Figure 5). We found that the expression of Wif1 decreased in the inhibitor group and increased in the activator group, while the expression of APC decreased in the activator group. PCP core genes (Prickle2 and Vangl2) did not change significantly in both groups. Interestingly, the expression of Axin2 was decreased in both groups, indicating that Axin2 may be an important role affecting the polarity of the utricle.

## 4. Discussion

The utricle is the organ that senses balance. Proper utricle function depends on the polarization of hair bundles located on the HC surface. This cellular polarization has also been found in many epithelial cell types, including in the respiratory airway and lateral ventricles of the brain. The planar polarity of the utricle was described at three levels: subcellular, cellular, and tissue-wide [8]. In the inner ear, tissue polarity exists only in the vestibular organ. HCs are oriented such that their stereociliary bundles point toward the LPR in the mammalian utricle. LPR was intact in mouse embryos at E15 [30]. Our previous studies found that the HCs in the lateral extrastriolar region (E13.5) appear later than in the medial extrastriolar regions (E11.5). Thus, E13.5 is a critical period for the formation of polarity [28]. In this study, we treated utricles at or before E13.5, either by adding inhibitor or activator *in vitro* or by knocking out *β*-catenin *in vivo*. The results showed that the canonical Wnt signaling pathway played a role in polarity development of the utricle.

Wnt signaling is a marker of embryonic development and has multiple functions at a number of developmental time points. The Wnt signaling pathway includes both canonical and noncanonical forms. The Wnt signaling pathway has been studied in the early development of the cochlea. The Wnt signaling receptors (Fzd 1, 2, 3, 4, 6, Ryk, Ror2, and Lgr5) can be detected in the cochlea of mice at E14.5 [31–36]. The expression of Wnt7a, Wnt5a, Wnt2, Wnt10b, Wnt4, Wnt7b, Wnt8, and Wnt11 could be detected in the cochlea at E15 and E17 [37], and studies suggested a potential alternation between canonical and noncanonical signaling pathways [38, 39]. Our study also revealed both canonical and noncanonical Wnt pathway-related genes in the utricle during early development, and most Wnt pathway genes were also different between the two time periods of E13.5 and P1. These greatly altered genes may contribute to valid proliferation, differentiation, or innervation. When LiCl was added to activate the Wnt pathway *in vitro*, the proliferation domain of Sox2-positive cells in the cochlea (E12.5) was significantly expanded, indicating that the canonical Wnt pathway regulates the proliferation and differentiation of HCs during early development of the cochlea [14]. Furthermore, overexpression of *β*-catenin initiated proliferation of sensory precursors cells within the cochlear sensory epithelium [40]. Therefore, we hypothesized that the Wnt pathway might play the same role in the early development of the utricle. This was confirmed *in vivo* when we conditionally knocked out *β*-catenin that the number of utricle HCs was affected in the mic. In addition, we found changes in the polarity of hair cells.

Indeed, the Wnt pathway also plays an important role in inner ear polarity regulation. The polarity of HCs in the cochlea is mainly reflected in their convergence and extension, an identical ciliary direction among all HCs, and a V-shaped stereociliary bundle with a uniform direction [41]. In vestibular organs, the stereocilia arrangement and kinocilium displacement are in accordance with a subcellular polarity. HCs are surrounded by supporting cells, and all HCs showed directional coordination in this study. Regarding tissue polarity, cilia bundles of HCs are present on both sides of the LPR face, or move away from the LPR. The role of the Wnt/PCP pathway, a noncanonical Wnt pathway, in inner ear polarity has been studied extensively [34, 42–44]. The noncanonical Wnt/PCP signaling pathway mainly involves Wnt proteins, such as Wnt4, Wnt5A, and Wnt11 [45]. These proteins activate the DEP domain of the Dsh protein, to in turn activate downstream homologous paleogenesis by PCP core proteins, independent of the accumulation of *β*-catenin. Dislocation of the hair bundle is a common phenotype of Wnt/PCP pathway mutants in the inner ear. The cochlea of the Wnt5a mutant is wider and shorter, with stereociliary bundle misorientation in accordance with a subcellular polarity [35]. After knocking out Vangl or Frizzled, which are the core PCP genes, we observed a disturbance in the polarity of neighbouring cells that appeared relatively consistent, while its subcellular polarity remained intact [34, 46]. In addition, Testin, Celsr1, and Pk2 affect the PCP protein distribution in the vestibule according to the cellular polarity [44, 47, 48]. It is generally believed that the PCP axis is established by noncanonical Wnt signals for tissue polarity. Therefore, it is worth investigating whether canonical Wnt signals play a role in the polarity of the utricle.

Our results showed that the Wnt genes expressed on different days in the utricle were closely related to canonical Wnt/*β*-catenin pathway, such as Axin2. Thus, we suspect that the canonical Wnt pathway also plays an important role in regulating polarity. The canonical Wnt pathway has been implicated in the regulation of polarity in other organs and animals. Local activation of the canonical Wnt pathway regulates neuronal polarity and axonal outgrowth [49]. In addition, our previous study found that Rack1 is involved in cell membrane localization of the polarity core protein Vangl2, which is associated with zebrafish planar cell polarity formation, primarily by antagonizing the Wnt/*β*-catenin signaling pathway [50]. *β*-catenin is the core protein in the canonical Wnt pathway that controls the expression of downstream target genes. A mouse with conditional knockout of *β*-catenin in the dorsal neural folds will exhibit spina bifida aperta, caudal axis bending, and tail truncation [51]. Similar polarity changes occurred in mouse kidneys with conditional knockout of *β*-catenin [52]. However, there are few studies on the canonical Wnt pathway in the context of polarity development in the inner ear, especially in the vestibule. Ankrd6 is asymmetrically distributed in the utricle and regulates polarity by inhibiting the canonical Wnt/*β*-catenin signaling pathway [53]. Some researchers suggested that the Wnt/*β*-catenin pathway is important in the ground hypothesis, which was suggested to explain the formation of LPR in the vestibule [54]; however, this has not been verified in mammals. Sox2 is expressed throughout the developing nervous system and plays a role in hair cell type formation [55]. We knocked out *β*-catenin in Sox2-positive cells and found that the utricle polarity was subsequently affected: the directions of some HCs were disordered near the LPR in the lateral and striolar regions. The changed polarity was also found after activating and inhibiting canonical Wnt pathways in vitro. The changed polarity was also found after activating and inhibiting canonical Wnt pathways in vitro, and PCP core genes (Vangl2 and Prickle2) were no statistical difference with IWP2 treatment or Licl treatment at the RNA level, compared with the control group. However, Axin2 unexpectedly decreased in the two treatment groups. Axin2, a scaffolding protein of glycogen synthase kinase 3, is a negative regulator of Wnt signaling. In addition, Axin2 feedback loop is important with many studies in Wnt pathway, which could help to limit Wnt-initiated signal [56]. Axin2 is involved in the development of the permanent teeth, hair, and eye brows and regulates calvarial suture closure in skull development [57, 58]. In the study of hair cells, a subset of hair cells is derived from Axin2-expressing tympanic border cells, and Axin2 cells were able to differentiate into hair cell-like cells [59]. These findings may suggest that Axin2 has the value in the polarity of utricle, which needs further study to elucidate.

## 5. Conclusion

In this study, we investigated expression changes of Wnt pathway-related genes during early development of the mouse utricle. In conditional knockout of *β*-catenin *in vivo*, our data showed the main effect is on hair cell number and may reveal an impact on stereociliary bundle orientation. Combined with the *in vitro* results, we believe that the canonical Wnt pathway is important for controlling HC polarity during mammalian utricle development. Our findings may stimulate future studies on vestibule polarity development.

## Data Availability

All data used to support the findings of this study are available from the corresponding author upon request.
